# Reactivity of observers’ facial skin blood flow depending on others’ facial expressions and blushing

**DOI:** 10.3389/fpsyg.2023.1259928

**Published:** 2023-12-07

**Authors:** Naoki Ishikawa, Masato Asahina, Satoshi Umeda

**Affiliations:** ^1^Graduate School of Human Relations, Keio University, Tokyo, Japan; ^2^Research Fellow of Japan Society for the Promotion of Science, Tokyo, Japan; ^3^Department of Neurology, Kanazawa Medical University, Ishikawa, Japan; ^4^Keio University Global Research Institute, Tokyo, Japan; ^5^Department of Psychology, Keio University, Tokyo, Japan

**Keywords:** facial skin blood flow, blushing, emotion, perception, anger

## Abstract

Facial skin blood flow (SkBF) has attracted attention as an autonomic indicator because it influences facial colour, which informs others of emotional states, and facial temperature related to social anxiety. Previous studies have examined the facial SkBF in people experiencing emotions; however, facial SkBF changes in the observers of emotions are poorly understood. Our study clarified facial SkBF changes related to observing others’ emotions by comparing the changes with other physiological indices. Thirty healthy participants (24 females; mean age: 22.17) observed six types of facial expressions (neutral, angry, and embarrassed expressions with and without facial blushing) and rated the emotional intensity of the other person. We measured their facial SkBF, finger SkBF, and cardiac RR interval as they made their observations. Facial SkBF generally decreased in relation to observing emotional faces (angry and embarrassed faces) and significantly decreased for angry expressions with blushing. None of the participants noticed blushing of facial stimuli. For the RR interval and finger SkBF, there was no variation depending on the observed facial expressions, although there was a general increase related to observation. These results indicated that facial SkBF is sensitive and reactive to emotional faces—especially angry faces with blushing— compared with other autonomic indices. The facial SkBF changes were not related to either RR interval changes or the intensity rating, suggesting that facial SkBF changes may be caused by vasoconstriction and have potential functions for our emotions. The decrease in facial SkBF may have a role in calming observers by preventing them from adopting the same emotional state as a person with intense anger. These findings clarify daily facial SkBF fluctuations and their relationship with our emotional processing in interpersonal situations.

## 1 Introduction

Psychological factors, such as emotions, induce fluctuations in human bodies during social interactions, including motor and autonomic nervous system (ANS) responses. Measuring autonomic responses in emotion studies is an effective method for understanding the relationship between ANS and emotions because the former fluctuates alongside the latter. Also, ANS responses are essential to the cognition of one’s emotional states (James, 1884; Schachter and Singer, 1962) through interoception (Sherrington, 1906). Typical autonomic responses include heart rate changes (Ekman et al., 1983; Bradley et al., 1990), sweating (Christopoulos et al., 2019), and tear production. However, recent studies have introduced facial skin blood flow (SkBF) as a new emotion-related autonomic index. Facial SkBF increases when experiencing emotions such as anger (Drummond, 1999; Drummond and Quah, 2001), joy (Drummond, 1994), anxiety (Hirota et al., 2017; Brummelman et al., 2018; Nikolić et al., 2018), and embarrassment (Drummond et al., 2003, 2007; Drummond and Bailey, 2013). Matsukawa et al. (2018) showed that the facial SkBF decreases when watching a comedy, a positively charged emotional stimulus. Hence, facial SkBF may generally increase during negative emotions, although there is no consensus on how positive emotions affect facial SkBF. Thus, facial SkBF is a critical index related to emotions.

Blood flow is related to skin colour (Hirota et al., 2017); therefore, facial SkBF could be related to blushing, which is biologically defined as the reddening or darkening (Leary et al., 1992; Thorstenson et al., 2020) of the face, ears, neck, and upper chest (de Melo et al., 2010). Blushing is assumed to occur because of vasodilation (Drummond, 1999; de Jong and Dijk, 2013) and cardiac output changes (Matsukawa et al., 2018). Blushing reportedly has social functions (Nikolić et al., 2016), implying that it is a signal to convey emotional information to observers. People believe that anger causes blushing (Drummond, 1997). Hence, they tend to judge facial redness as an angry facial expression (Nakajima et al., 2017). Moreover, people interpret blushing as reflection or sincerity (Dijk et al., 2011; Thorstenson et al., 2020). Changes in facial SkBF also reflect facial temperature changes (Shearn et al., 1990), which leads to social anxiety because of the discomfort brought about by facial warmth. The fluctuation in facial temperature has been investigated as a similar autonomic indicator of facial SkBF (Ioannou et al., 2014a). Thus, facial SkBF is a unique autonomic index that involves changes in facial colour and temperature.

In social interactions, we can be both experiencing emotions and observing others’ emotions. Observers estimate others’ emotions by referring to emotional signals expressed by the latter (Preston and de Waal, 2002)—such mental activity is called empathy (Umeda, 2012). These signals include facial muscle activity and tearing as motor (Ekman and Friesen, 1976) and autonomic (Vingerhoets et al., 2016) responses, respectively. In addition to conscious signal perception, observers estimate others’ emotions through unconscious signal perception, as in the example of the pupillary response (Harrison et al., 2006). Besides the cognitive aspect, it is important to focus on the physical reactions of emotional observers during these interactions. For example, an observer who perceives a smile from others also smiles, a phenomenon called facial mimicry or motor synchrony (Likowski et al., 2012; Prochazkova and Kret, 2017). This synchrony may play a role in understanding others’ emotions as both parties—the observer of the emotion and the one experiencing it—assume the same state (Kret, 2015).

Another example would be how angry expressions are threat-related signals (Leppänen et al., 2018). Observing a person with an angry facial expression may increase the observer’s heart rate. In a series of interpersonal interactions, observers’ reactions are fed back to the original person experiencing emotions and, thus, might alter their emotional experience.

While studies have shown that facial SkBF is related to emotions, the facial SkBF dynamics related to observing others experiencing emotions are poorly understood. Focusing on the physiological responses of the observer is important in the context of emotional interactions (Prochazkova and Kret, 2017). Therefore, we focused on the observers’ facial SkBF changes related to observing the others’ signals. Since facial SkBF is related to facial colour as an emotional signal, measuring observers’ facial SkBF could clarify the emotional interaction (accompanied by facial colour) from observers to the original person experiencing emotions. This study revealed the facial SkBF changes associated with the observation of others’ emotions consisting of a combination of facial expressions (neutral, angry, or embarrassed) and facial blushing (gradually blushing or no blushing) through comparison with cardiovascular indicators, such as RR interval and finger SkBF. Finger SkBF, which is the same blood flow index as facial SkBF, reflects sympathetic activity levels (Kistler et al., 1998), and several studies have often reported a discrepancy between facial and finger SkBF. Therefore, we measured finger SkBF to highlight the specific reactivity of facial SkBF by examining the deviation from the finger SkBF. RR interval is the index related to cardiac activity, and the interval reflects the level of autonomic activity. In addition, the cardiac output can influence facial SkBF fluctuations, and the cardiac output can be estimated to some extent from changes in heart rate. We measured the RR interval to estimate whether facial SkBF fluctuations were caused by vasomotor responses or blood circulation originating from the heart.

The purpose of this study was to examine an observer’s facial SkBF related to observing the emotional signals sent by facial expressions and blushing. We hypothesised that facial SkBF would be more sensitive than other physiological indicators and increase depending on others’ emotional expressions (angry and embarrassed expressions with blushing), which is physiological synchrony.

## 2 Materials and methods

### 2.1 Participants

We recruited 6 male and 24 female undergraduate participants aged 19–25 (mean = 22.17 years, standard deviation = 1.32) with normal visual acuity and colour vision. None of the participants had epilepsy, neurosurgical history, or diseases that could affect the measurements of physiological indicators. Participants were instructed not to use makeup or consume vasoactive substances, such as alcohol, nicotine, or caffeine, 3 h before the experiment. Participants took part in the experiment between November and early December, which is fall or winter in Japan.

The Research Ethics Committee of the Faculty of Letters, the Graduate School of Faculty, and the Graduate School of Social Science of Keio University approved this study (190220000). We conducted this experiment according to the principles of the Declaration of Helsinki. Participants provided written informed consent after being briefed about the experimental protocol. After completing the experiments, participants were paid 3,000 Japanese Yen for their participation.

### 2.2 Apparatus

**Figure 1:**
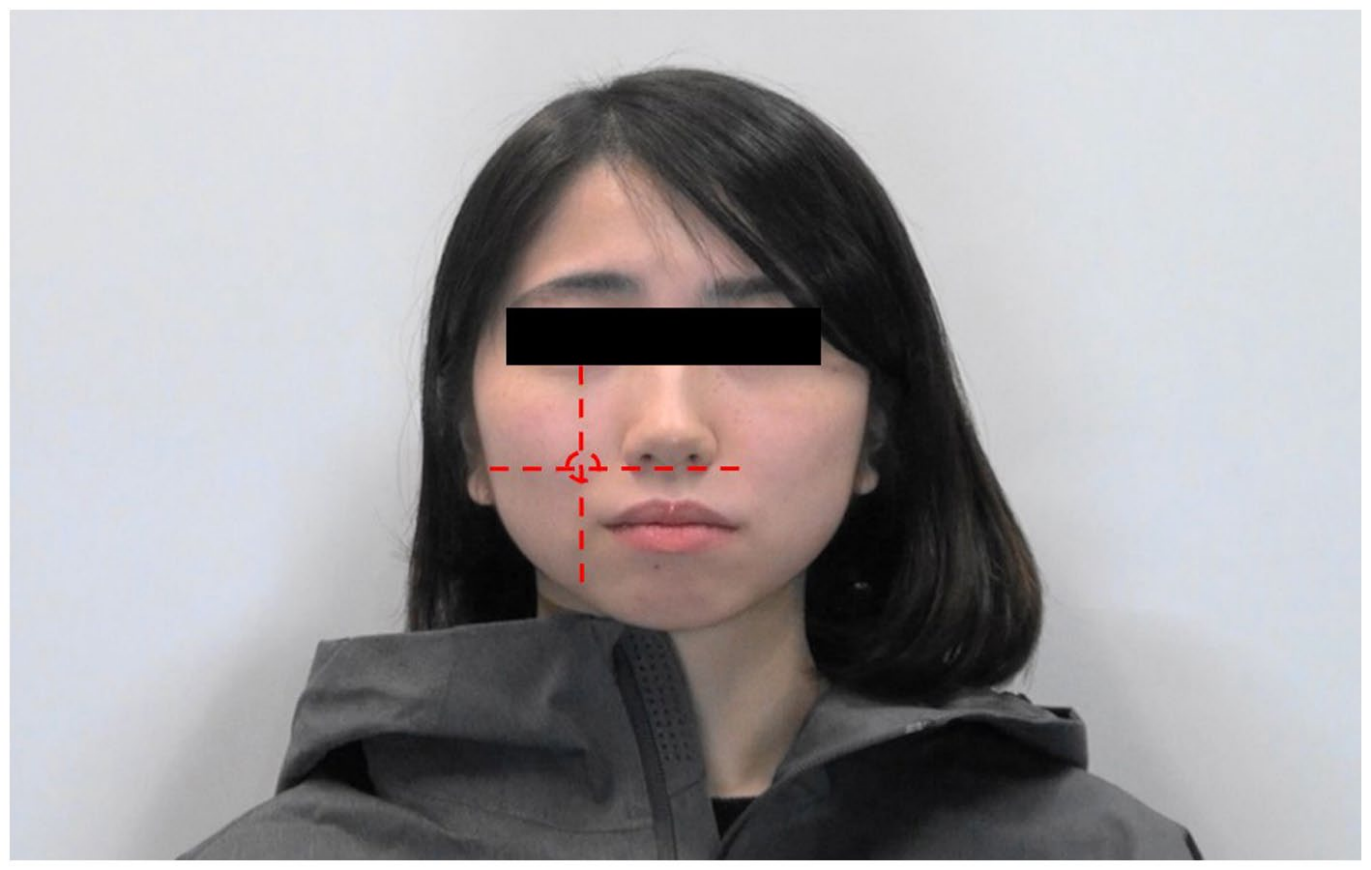
Area of the face where the pulse transducer is attached. The red circle indicates the region where the transducer is attached.

We used a pulse-measuring amplifier (PPG100C, Biopac System, Inc.) to measure the RR interval. A pulse transducer (ALF21D, Advance) was used to detect facial and finger SkBF changes. The sample rate for these devices was 2000 Hz. The transducer can be attached to various face positions (Ioannou et al., 2014a). Since several studies have focused on the cheek area (Shearn et al., 1992; Drummond, 1994; Brummelman et al., 2018; Matsukawa et al., 2018), we attached the transducer directly on the right cheek at the intersection between the vertical line drawn from the centre of the pupil and the horizontal line drawn from the top of the philtrum (Figure 1).

Additionally, a transducer was placed on the participant’s right index finger on the palmar side to assess finger SkBF. Participants took part in the experiment individually, where the mean temperature was 24.6 ± 1.2°C. The mean humidity was 36.3 ± 3%. No participant felt either cold or hot during the test.

### 2.3 Stimulus

**Figure 2:**
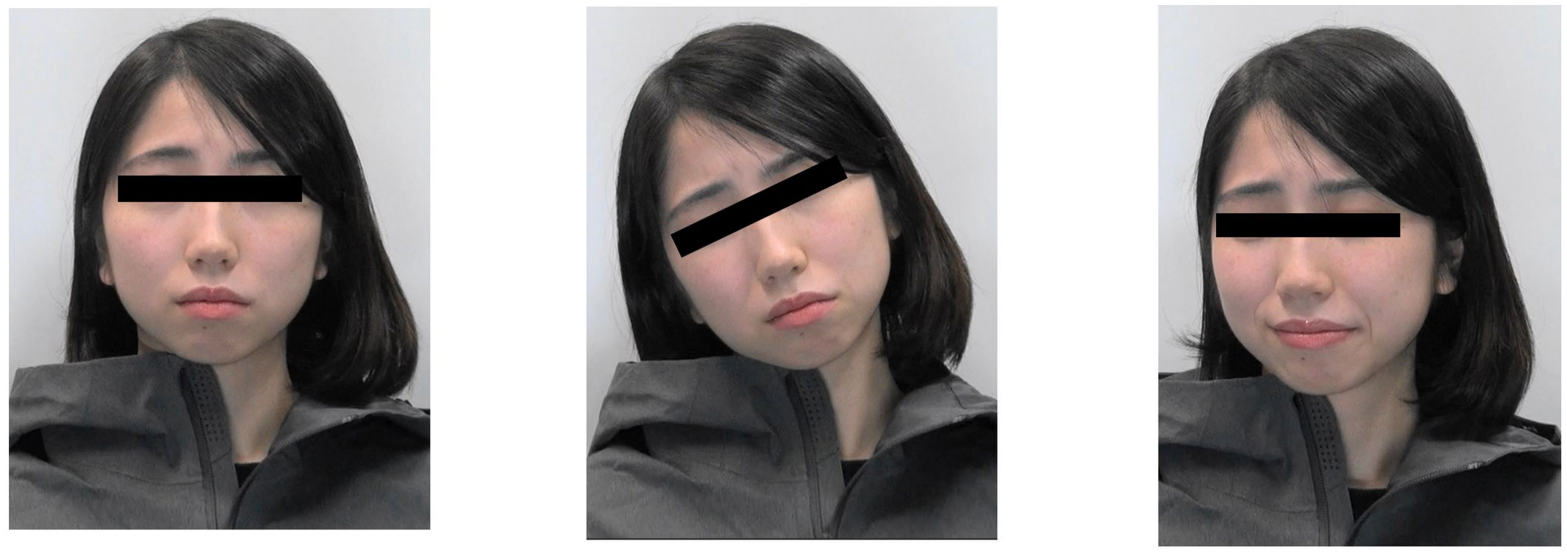
Examples of facial expressions of a model displayed in this experiment. The one on the left is neutral, the one on the middle is angry, and the one on the right is embarrassed.

We documented 5-s video recordings of the models’ responses. In this video, five models (three females) made three facial expressions (neutral, angry, and embarrassed). We focused on angry and embarrassed expressions as emotional stimuli because anger and embarrassment have been proven to consistently induce an increase in facial SkBF, and there are natural associations with blushing. The angry expressions were based on the findings of Ekman and Friesen (1976), while the embarrassed expressions were based on the findings of Lewis et al. (1989) and Ioannou et al. (2017). In angry expressions, models were instructed to shut their mouths and wrinkle their brows. In embarrassed expressions, models were instructed to turn their gaze downward (straight down or diagonally down), and slightly raise the zygomaticus, but not smile open-mouthed (Figure 2).

We edited the 5-s video of 120 frames using the open-source software GNU Image Manipulation Program (GIMP; Version 2.10.0) to create blushing stimuli. Four parameters were used for editing: colour, maximum value, region, and frame window.

**Figure 3:**
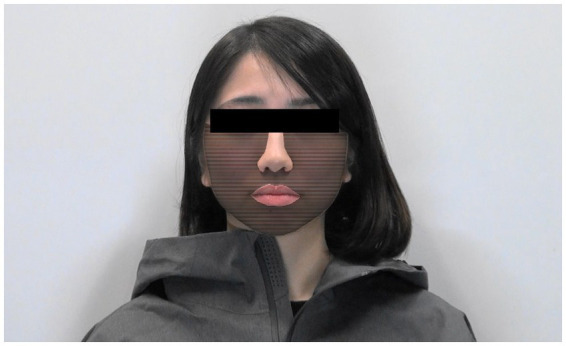
Edited region. The shaded area is the edited region.

**Figure 4:**
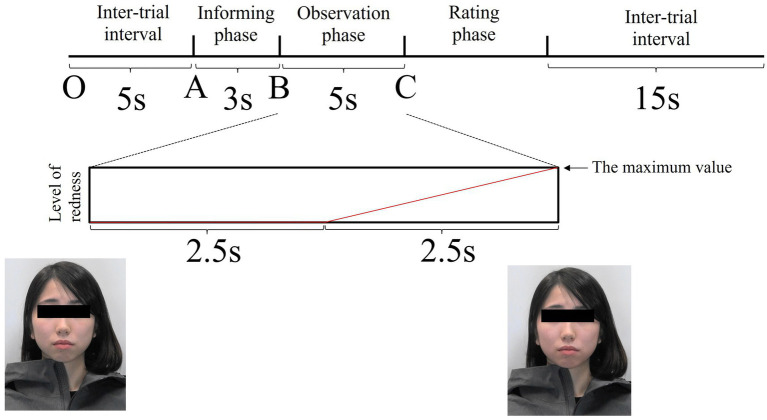
The trial structure. The model (a combination of colours × facial expressions) was displayed for 5 s. The image shown at Point C is maximally coloured red. The red line is an image of the increase in facial redness in the blush stimulus. The horizontal axis represents time, and the vertical axis represents the level of redness.

For colour, we used lip colour for each model image. For the maximum value, we adopted a value just below the threshold at which five additional participants perceived colour changes for each model. In daily life, it is difficult to notice facial colour changes. Therefore, it is necessary to investigate whether the unnoticed colour consciously affects observers, and we operated facial colour at a level that participants overlooked. For the region, we targeted the cheek area (Figure 3). For the frame window, we coloured 60 frames in the second half of the 120 frames. The last frame value of the 60 frames was set to the maximum value, and the colour was added evenly, starting from the first frame (Figure 4). From frames 61 to 120, the a*(CIE L*a*b*) value representing redness increased by an average of 1.73 as measured by Adobe Photoshop. Two types of editing were performed: gradual facial colour change and no colour change. Three facial expressions and two colour changes were obtained from the five models, resulting in 30 videos. Each stimulus was presented four times in random order; thus, each participant underwent 120 trials.

### 2.4 Procedure

Participants were instructed to sit up straight on a chair and look at the face of the person appearing on the display in front of them. Participants were required to rate the emotional intensity of the models for each trial from 1 to 7 using their left hand and to decide on the score during the rating phase (i.e., not before the observation phase offset). Participants were asked to be still as much as possible to limit movement artefacts while SkBF was being recorded. Additionally, an experimenter emphasised to the participants that the models’ emotions were not directed toward them.

Previous studies on blood flow have reported that participants usually take approximately 10 to 20 min to acclimatise to the indoor temperature (Ioannou et al., 2014a). The participants in our study were left for approximately 10 min in the experimental space to acclimatise to the indoor temperature before the experiment. Thereafter, the instruments to measure the physiological parameters (facial and finger pulse transducer and pulse-measuring amplifier) were attached to the participants. To avoid blood flow obstruction, the experimenter asked the participants if the tape fit tightly around their right index fingers. Each time the participant reported such tightness, the experimenter adjusted the transducer fit. After setting up the instruments, the participants were left alone for 5 min. In total, our experiment afforded participants a 15 min acclimatisation period. In addition, participants took a 5-min rest break after the first 60 trials were recorded. After the experiment, the instruments were detached, and the participants answered whether they had noticed blushing in the facial stimuli.

Each trial consisted of three phases (Figure 4). Phase 1 was the informing phase, in which the neutral facial expression of a model was displayed for 3 s, and a word that incited a specific emotion in the model was sounded simultaneously. In this phase, participants discovered that the model was told words that would cause a particular emotion. If the neutral expression was to be displayed in the next phase, then the participants randomly heard, in the informing phase, words like ‘You are right-handed, aren’t you?’ or ‘You get up early, do not you?’ If the angry expression was to be displayed in the next phase, the words the participants heard were ‘Please stay away from me’ or ‘Why do not you make a little effort?’ If the embarrassed expression was to be displayed in the next phase, the words the participants heard were ‘Your test score was bad, wasn’t it?’ or ‘You fell on the street a while ago.’ We did not present emotional labels to improve ecological validity. Phase 2 was the observation phase, wherein a combination of colours (gradually blushed or no change) and facial expressions (neutral, angry, or embarrassed) were randomly presented for 5 s. The last phase was the rating phase, during which the participants rated the emotional intensity the models presented in the observation phase on a scale of 1 to 7 (weak to strong, respectively) within approximately 5 s. There was a 15 s inter-trial interval between the end of the rating phase and the onset of the next informing phase, during which the participants were provided a fixation point to concentrate on.

### 2.5 Data on physiological and subjective indices

The AcqKnowledge software recorded the physiological responses through an A/D converter (MP150, Biopac System, Inc.). Additionally, the onset and offset of each phase were recorded using a stimulus presentation interface (STK100, Biopac System, Inc.).

We set baseline and target time windows for all physiological responses, as described below. The mean values during the baseline and target windows were defined as the baseline and target values, respectively.

**Figure 5:**
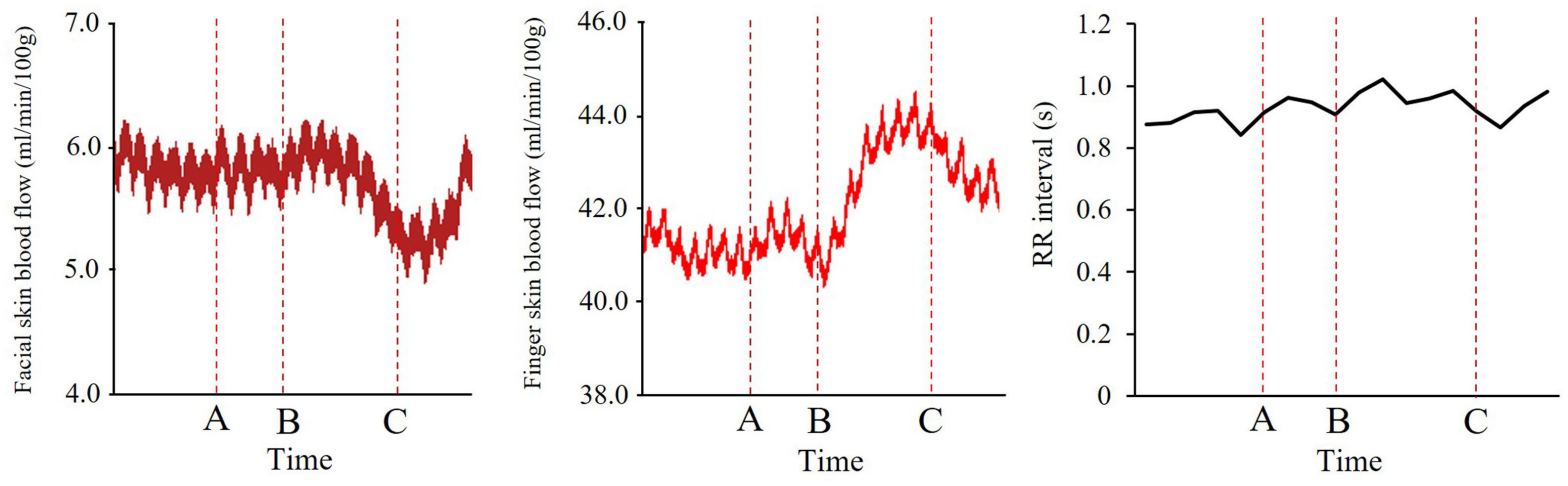
Examples of time course data in each physiological index. The labels A–C correspond to the structure of the trial in Figure 4. From left to right are facial SkBF, finger SkBF, and RR interval.

In the facial and finger SkBF analyses, baseline time windows were set as 5 s before the onset of the informing phase (between O and A; Figure 4). Baseline windows for RR-interval were set as 5 intervals before the onset of the informing phase. For facial and finger SkBF, we defined the target time windows as 2.5 s before and after the end of the stimulus presentation (5 s in total). The 2.5 s before the end of the stimulus presentation included the blush change, and the 2.5 s after the end of the stimulus were considered to be the time that could be affected by the previous stimulus. Participants were asked to rate the emotional intensity after observing others’ emotions in this experiment. However, we did not tell them to rate it in a hurry; therefore, the 2 or 3 s after the observation was expected to be devoid of a response to the rating. In the RR interval, the target window was set as a total of five intervals across the end of the stimulus presentation. Figure 5 shows examples of the changes in each physiological index (facial SkBF, finger SkBF, and RR interval).

Subsequently, the change rate for each trial was calculated according to the following formula:


$$\left\{\left[\left(\mathrm{target}\;\mathrm{value}-\mathrm{baseline}\;\mathrm{value}\right)/\mathrm{baseline}\;\mathrm{value}\right]\times 100\right\}$$

The change rate for 120 trials was calculated for each physiological index and each participant, and subsequent analyses were performed using this change rate. We then took the average change rate within each combination (2 colours × 3 facial expressions) to produce the representative value for each of the six types of faces. Finally, similar to the physiological indices, we obtained the subjective rating values for each trial and their means within each combination. In situations where people observe others with emotions, observers generally estimate the emotional intensity and the type of emotions of others. We focused on the quantitative intensity rating to analyse the relationship between the change in facial SkBF and the subjective rating. On the other hand, rating the types of the models’ emotions was verbally conducted at the end of the experiment, and we confirmed that all participants discriminated between neutral, angry, and embarrassed expressions.

### 2.6 Statistical analysis

Previous studies used parametric tests, such as analysis of variance (ANOVA) and Pearson’s correlation test, to analyse the SkBF and heart rate (Drummond and Su, 2012; Drummond and Bailey, 2013; Kashima et al., 2013). We used two-way repeated measures ANOVA to examine the effect of blushing and facial expressions on physiological and subjective indices.

In facial SkBF, which is the focus of this study, normality in the Shapiro–Wilk test has been confirmed. The factors were colour (blush or non-blush) and facial expressions (neutral, angry, or embarrassed). If ANOVA showed interactions between factors (colour and facial expressions), we used a simple main effects test with Bonferroni correction. In addition to comparing conditions, we used a one-sample *t*-test for all physiological indices to clarify whether physiological fluctuations occurred or if an observed fluctuation was significant compared with the baseline values. We studied the responsiveness of facial SkBF to emotional faces by comparing the results of ANOVA and one-sample t-test across the physiological indices.

We further analysed the correlation between the change rate of facial SkBF and RR interval for each combination to examine whether a vasomotor response caused the fluctuations in facial SkBF associated with observing others’ emotions. We also performed a correlation analysis using Pearson’s correlation coefficient between the changes in facial SkBF and subjective rating to examine this relationship.

All statistical analyses were performed using SPSS software (IBM SPSS Statistics for Windows, Version 25.0, Armonk, NY: IBM Corp.). We excluded values deviating by more than two standard deviations in all analyses. In addition, if it was clear that the body movements affected the physiological measurements, the trial in question was excluded. The analysis using Morepower (Campbell and Thompson, 2012) showed that the sample size was sufficient for the interaction between colour and facial expressions on facial SkBF, described below.

## 3 Results

### 3.1 Physiological indices

#### 3.1.1 Facial SkBF

**Figure 6:**
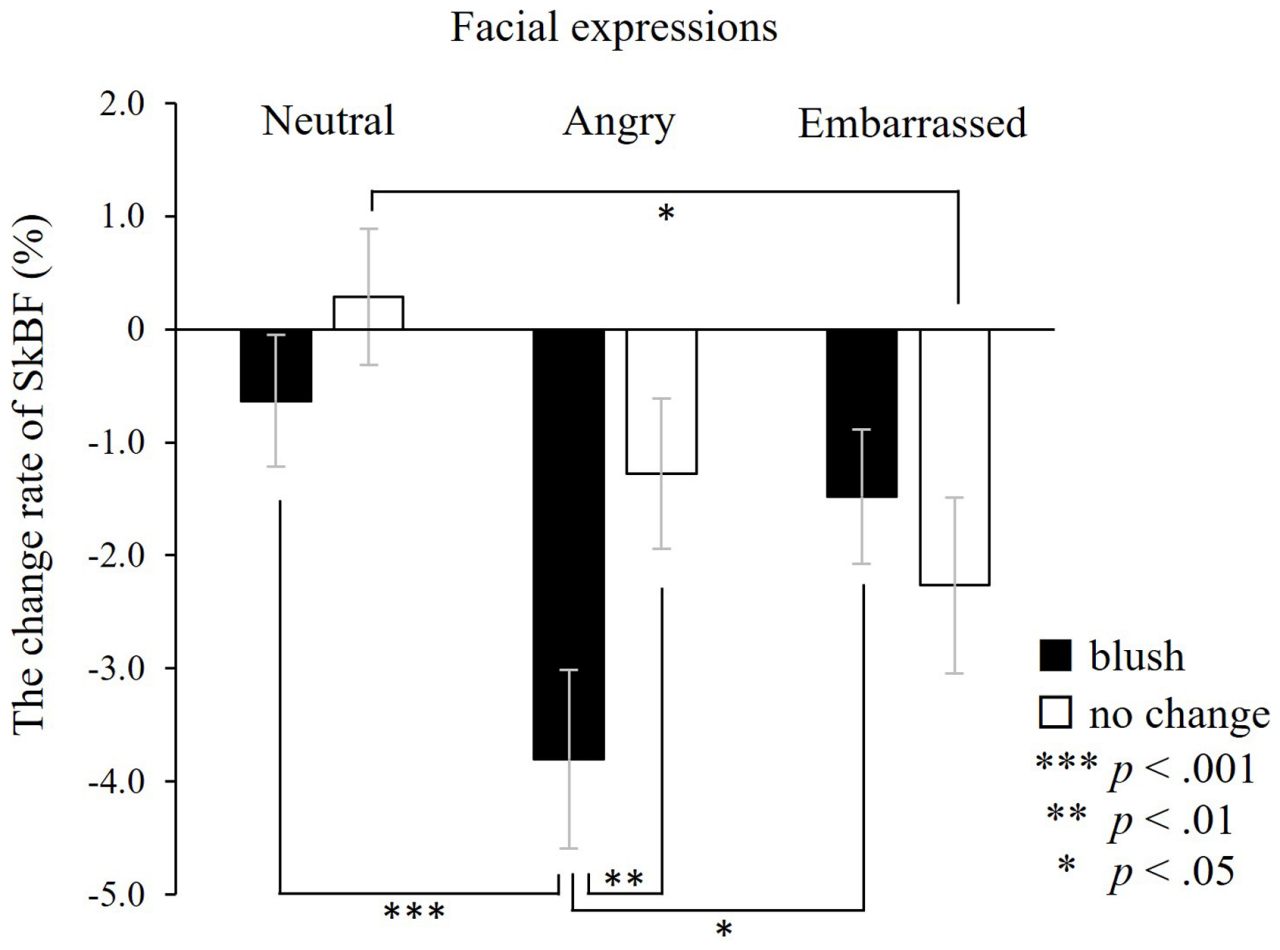
The change rate of facial skin blood flow (SkBF) in the target window. Facial SkBF decreases significantly associated with observing angry expressions with blushing compared with angry expressions without blushing, neutral expressions with blushing, and embarrassed expressions with blushing. ***p < 0.001, **p < 0.01, and *p < 0.05. The error bar represents the standard error.

ANOVA was used to investigate changes in facial SkBF during the target window (Figure 6). The analysis showed a significant interaction between colours and facial expressions (*F*(2, 42) = 6.50, *p* < 0.01). Also, the simple main effects test with Bonferroni correction indicated that facial SkBF decreased when participants observed angry expressions with blushing, compared with angry expressions without blushing, neutral expressions with blushing and embarrassed expressions with blushing.

The main effects of facial expressions were significant (*F*(2, 42) = 9.22, *p* < 0.001), while colour was not (*F*(1, 21) = 3.86, n.s.). The one-sample *t*-test performed to examine whether SkBF changed from baseline indicated that facial SkBF decreased significantly only in faces associated with anger and embarrassment (with blushing × neutral: *t*(26) = −0.62, n.s.; angry: *t*(26) = −4.64, *p* < 0.001; embarrassed: *t*(26) = −2.20, *p* < 0.05; without blushing × neutral: *t*(26) = 0.91, n.s.; angry: *t*(26) = −2.68, *p* < 0.05; embarrassed: *t*(26) = −2.84, *p* < 0.01).

These results showed that facial SkBF decreased significantly in all types of faces associated with emotional facial expressions. Moreover, facial SkBF had the most significant decrease when faced with angry facial expressions with blushing.

#### 3.1.2 Cardiac activity

**Figure 7:**
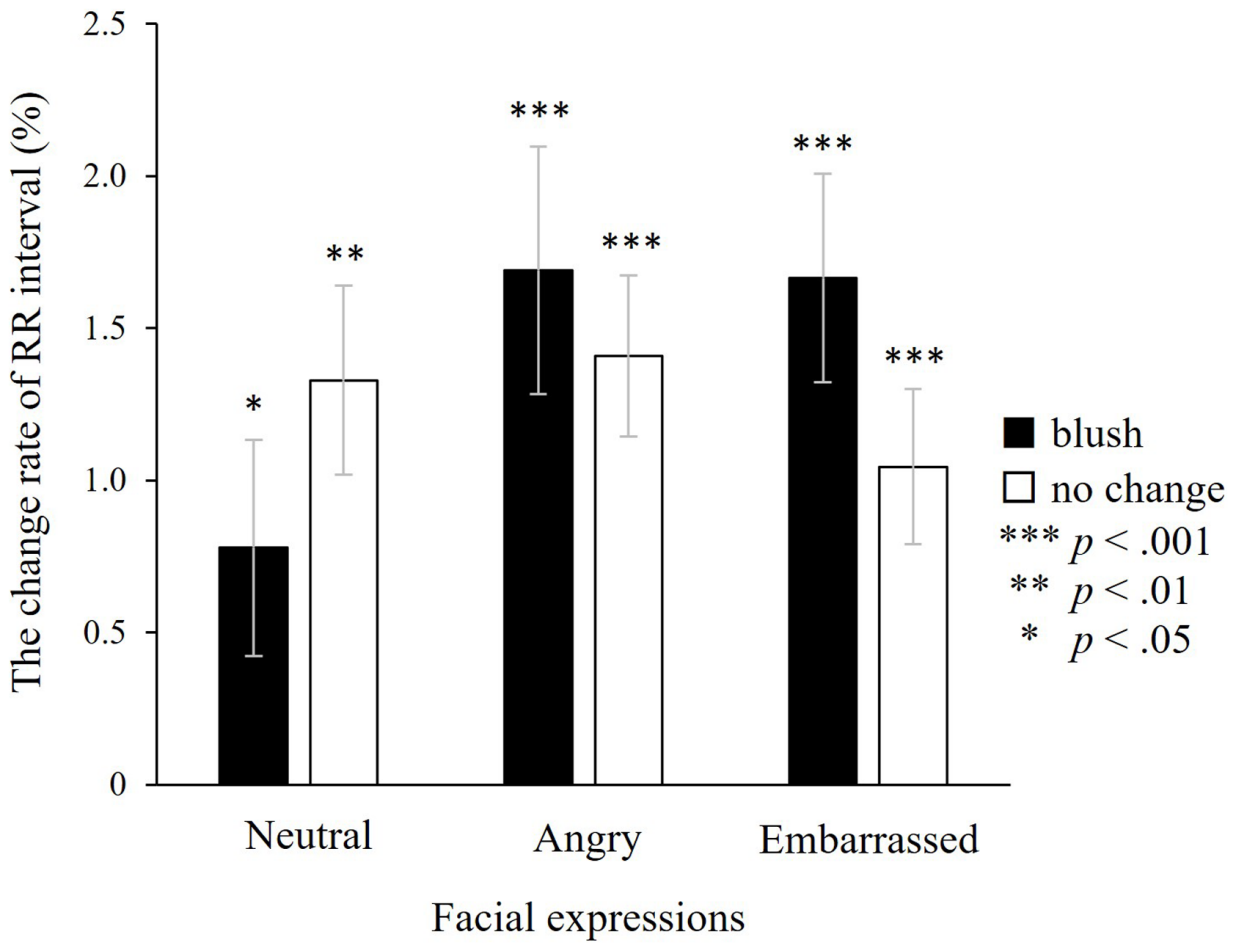
The change rate of the RR interval in the target window. An increment in the RR interval indicates heart rate reduction. There are no significant effects, indicating that the RR interval does not fluctuate in relation to colour or facial expressions. However, a one-sample *t*-test indicates a significant increase in the RR interval, indicating that the heart rate slows down in all types of faces, irrespective of colour change or facial expression. ***p < 0.001, **p < 0.01, and *p < 0.05, which indicate significant increases from baseline. The error bar represents the standard error.

The RR interval was investigated using ANOVA during the target window. There were no main effects of colour and facial expressions and no significant interaction between colours and facial expressions (colours: *F*(1, 24) = 0.22, n.s.; facial expressions: *F*(2, 48) = 1.16, n.s.; colours × facial expressions: *F*(2, 48) = 2.98, n.s.; Figure 7). The *post hoc* one-sample *t*-test demonstrated that the RR interval increased significantly for all types of faces (with blushing × neutral: *t*(27) = 2.55, *p* < 0.05; angry: *t*(27) = 3.97, *p* < 0.001; embarrassed: *t*(27) = 4.09, *p* < 0.001; without blushing × neutral: *t*(28) = 3.55, *p* < 0.01; angry: *t*(27) = 4.54, *p* < 0.001; embarrassed: *t*(27) = 3.83, *p* < 0.001).

#### 3.1.3 Finger SkBF

**Figure 8:**
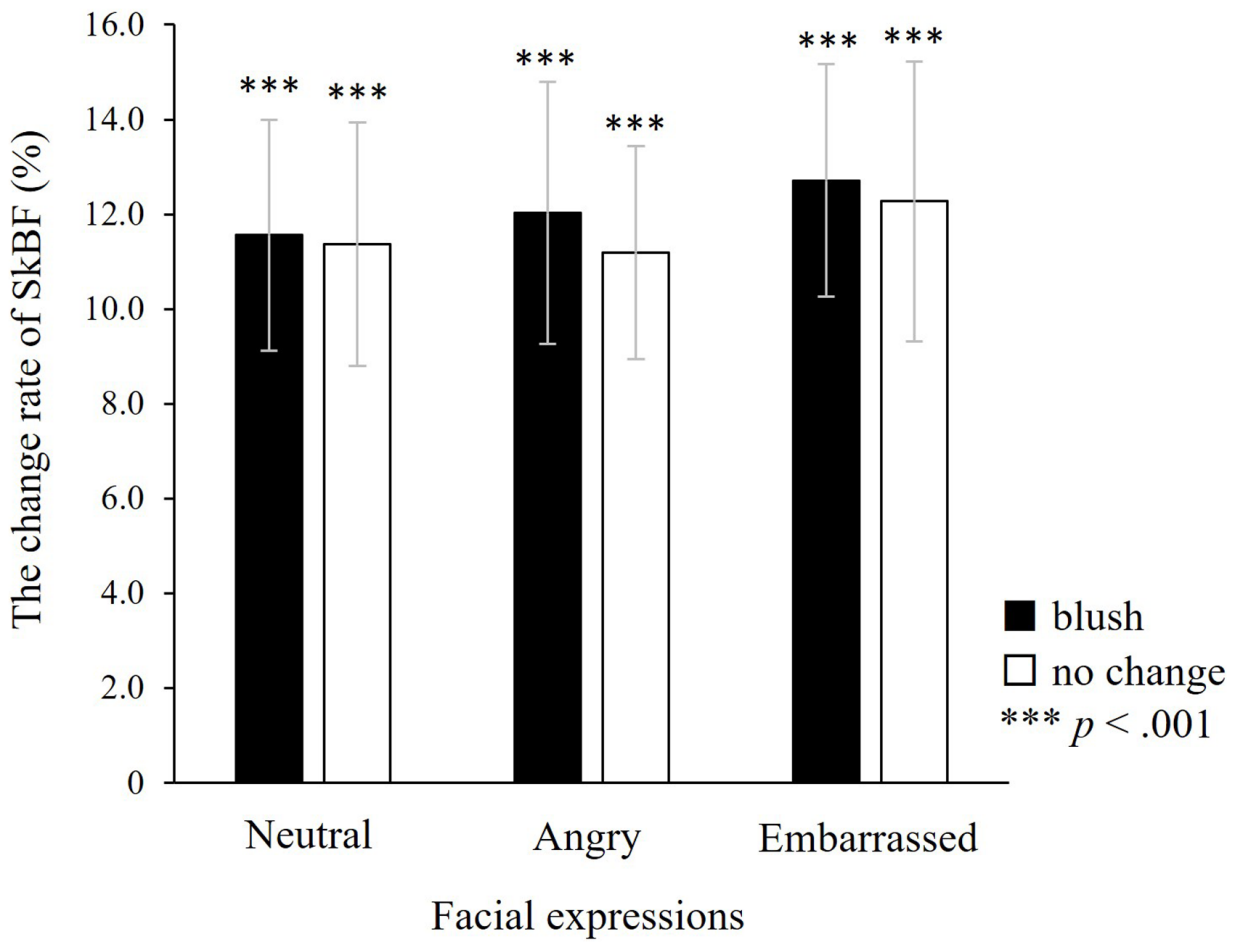
The change rate of finger skin blood flow (SkBF) in the target window. There are no significant effects, indicating that finger SkBF does not fluctuate in relation to colour or facial expressions. However, a one-sample *t*-test indicates that finger SkBF significantly increases in all faces, irrespective of colour change or facial expression. ***p < 0.001, which indicates significant increases from baseline. The error bar represents the standard error.

ANOVA was used to investigate the changes in peripheral SkBF during the target window. There were neither main effects of colour and facial expressions nor significant interaction between colours and facial expressions (colour: *F*(1, 25) = 0.12, n.s.; facial expressions: *F*(2, 50) = 0.12, n.s.; colours × facial expressions: *F*(2, 50) = 0.01, n.s.; Figure 8). A *post hoc* one-sample *t*-test demonstrated that finger SkBF increased significantly for all types of faces compared with baseline (with blushing × neutral: *t*(26) = 5.03, *p* < 0.001; angry: *t*(26) = 4.16, *p* < 0.001; embarrassed: *t*(26) = 5.38, *p* < 0.001; without blushing × neutral: *t*(27) = 4.93, *p* < 0.001; angry: *t*(27) = 5.39, *p* < 0.001; embarrassed: *t*(26) = 4.30, *p* < 0.001).

#### 3.1.4 Correlation between facial SkBF and cardiac activity related to facial observations

To clarify whether the heart rate affected the decrease in facial SkBF during the target window, we analysed the correlation between facial SkBF and the RR interval for each face type. We found no significant correlations for all types of faces (with blushing × neutral: *r* = 0.02, n.s.; angry: *r* = −0.08, n.s.; embarrassed: *r* = −0.34, n.s.; without blushing × neutral: *r* = −0.03, n.s.; angry: *r* = −0.19, n.s.; embarrassed: *r* = −0.02, n.s.).

### 3.2 Subjective index

**Figure 9:**
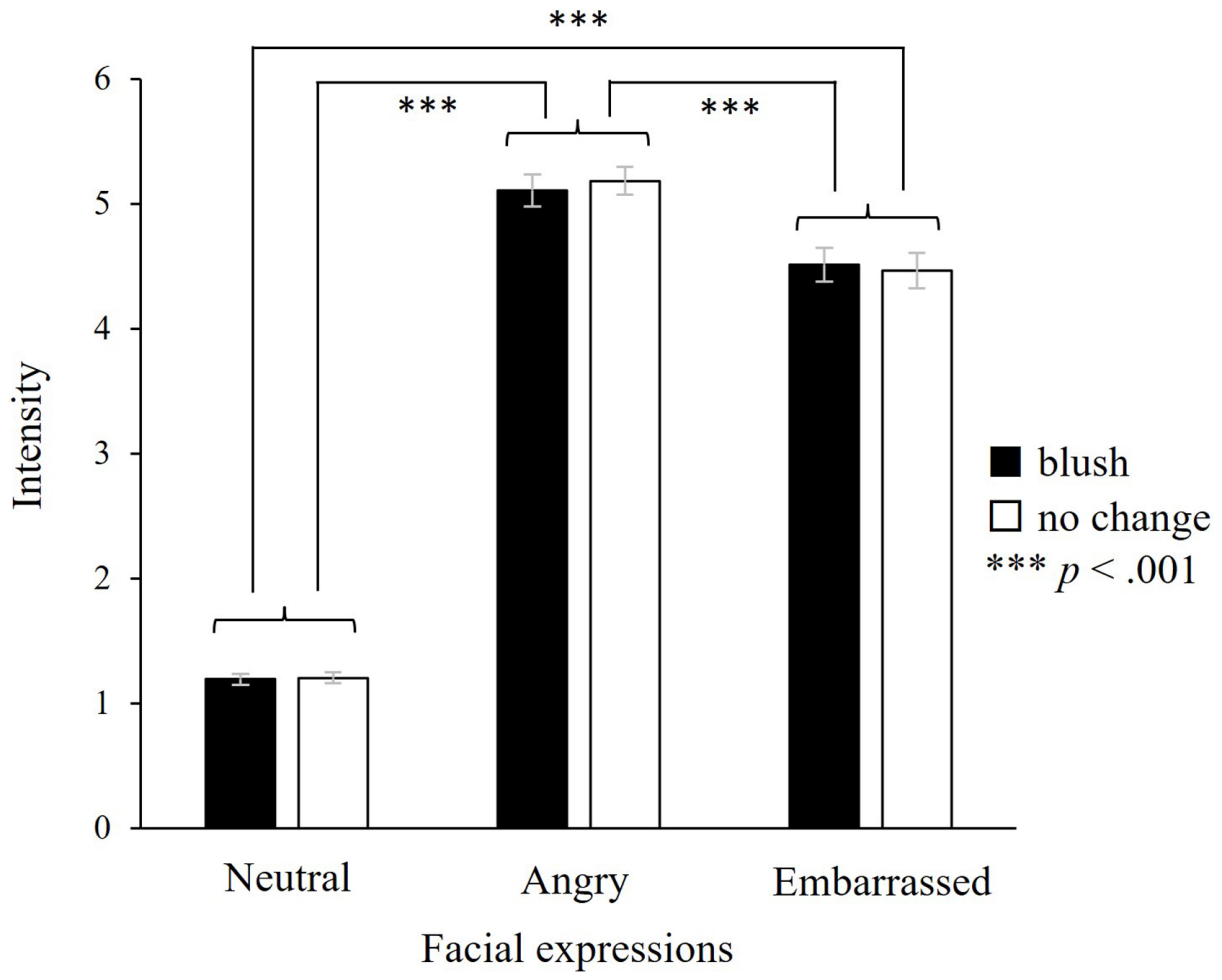
Intensity rating for each type of face. There is a significant main effect of facial expressions, indicating that angry expressions are rated the highest, followed by embarrassed and neutral expressions, successively. However, there are no significant main effects of colours or interaction between colours and facial expressions, showing that our facial expressions are valid and colour change does not influence the subjective rating. Participants rated emotional intensity from 1 to 7. ***p < 0.001, and the error bar represents the standard error.

Our results indicated a significant main effect of facial expressions (*F*(2, 46) = 588.49, *p* < 0.001; Figure 9) on intensity rating. Multiple comparisons showed that the ratings for angry, embarrassed, and neutral expressions were the highest, second highest, and lowest, respectively. Based on the differences between neutral expressions and the other two expressions, the facial expressions in our study were considered valid empathic stimuli. There were no significant main effects of colour or interaction between colours and facial expressions (colours: *F*(1, 23) = 0.48, n.s.; colours × facial expressions: *F*(2, 46) = 2.65, n.s.). All 30 participants reported that they were not aware of facial colour changes. Thus, the physiological indices did not reflect the awareness of colour change.

### 3.3 Correlation between facial SkBF and subjective ratings

We performed the correlation analysis to clarify the relationship between facial SkBF related to observing emotional faces and subjective ratings. However, we found no significant correlations for all types of faces (with blushing × neutral: *r* = 0.08, n.s.; angry: *r* = 0.03, n.s.; embarrassed: *r* = −0.15, n.s.; without blushing × neutral: *r* = 0.04, n.s.; angry: *r* = −0.12, n.s.; embarrassed: *r* = −0.31, n.s.).

## 4 Discussion

### 4.1 Change in facial SkBF related to observing a combination of facial expressions and blushing

This study aimed to examine an observer’s facial SkBF related to their observation of emotional signals sent by others’ facial expressions and blushing. Subjective reports on whether participants noticed blushing in facial stimuli and the subjective rating for emotional intensity showed that participants perceived facial expressions explicitly and experienced facial blushing implicitly.

We hypothesised that facial SkBF would be more sensitive than other physiological indicators and increase depending on the observed emotional expressions (angry and embarrassed expressions with blushing), which is physiological synchrony. However, the facial SkBF dynamics we derived were opposite of what was expected.

Our results showed that facial SkBF generally decreased associated with observing emotional expressions (angry expressions with or without blushing and embarrassed expressions with or without blushing) and noticeably decreased when faced with angry expressions with blushing compared with those in angry expressions without blushing, neutral expressions with blushing, and embarrassed expressions with blushing.

There are several possible reasons why facial SkBF decreased, especially in angry facial expressions with blushing. First, as Creavy et al. (2020) showed, the significant decrease is to maintain an emotional balance between the observer and the person experiencing intense anger— the observer calms down, in contrast to the person who has an angry expression with blushing. The RR interval in our results, indicating that the heart rate decreased associated with facial observation, partially supported the first reason. In addition, if the decrease in the observer’s facial SkBF induces facial colour change (by causing facial pallor), further interaction can occur between the observer with facial pallor and the person displaying intense anger, such as reducing the latter’s anger intensity.

Second, facial SkBF might have decreased because participants were scared by angry expressions with blushing. However, our analysis involving the RR interval did not coincide with this. The RR interval increased in all types of faces, indicating that the heart rate generally decreased. Since the heart rate increases during a state of fear (Rainville et al., 2006), we inferred that our study participants were not afraid of angry expressions.

Third, a decreased facial SkBF in response to observing angry expressions with blushing may reflect a large emotional processing load. The subjective rating showed that the processing load for angry expressions was larger than that for the other expressions. In addition to angry expressions, there was a load related to blushing, and these stacked loads might appear in the significant decrease in facial SkBF. However, our correlation analysis between the decreased facial SkBF and emotional rating did not support this third possibility.

Fourth, we focused on the nature of angry expressions. Anger is one of the basic emotions (Ekman, 1992), and it requires an immediate coping mechanism on the recipient’s part (LeDoux, 1996). As such, the decrease in facial SkBF may be a response specialising in the perception of angry expressions. The suggestion that facial blood flow decrease reflects a reallocation of blood flow to motor muscles in preparation for fighting or fleeing (Hare and Blevings, 1975) supports our fourth interpretation. Facial SkBF changed rapidly in response to a combination of a specific facial expression and implicitly perceived blushing. Therefore, our study findings highlight the importance of facial SkBF as an emotional indicator.

### 4.2 Relationship between facial SkBF and the other physiological indices

We measured finger SkBF and the RR interval in addition to facial SkBF related to observing others’ emotions. Continuous blood pressure measurement is needed to accurately evaluate vasodynamics. Nevertheless, our RR interval findings provide insight into the physiological mechanism of facial SkBF in response to observing the emotions of others. Generally, vasomotor reactions or heart rates influence facial blood flow (Ioannou et al., 2014b). The correlation analysis, which indicated no significant correlations between the RR interval and facial SkBF for all types of faces, suggested that the SkBF decrease in response to angry expressions with blushing could be caused by facial vasoconstriction rather than changes in heart rate.

Furthermore, we focused on the RR interval and finger SkBF. There were no significant differences in RR interval depending on the type of face; moreover, the interval generally increased, indicating that the heart rate decreased, irrespective of the type of face. Berntson et al. (1996) and Jönsson and Sonnby-Borgström (2003) reported that the heart rate decreases during attention to stimuli because of vagal nerve enhancement. Also, the act of observation may have reduced the fine movements of the body and decreased the heart rate. In our study, no specific pattern corresponded to emotions, and thus, the RR interval reflected the general attention paid to the models’ faces.

Like the RR interval, finger SkBF showed no effect related to the types of faces, and SkBF increased for all face types. Previous studies that focused on dissociating facial and finger SkBF while performing some tasks mainly reported that the increase in facial SkBF was accompanied by a decrease in finger SkBF (Drummond, 1994; Drummond and Quah, 2001; Vassend and Knardahl, 2005). Consistent with those findings, our study demonstrated a decrease in facial SkBF accompanied by an increase in finger SkBF associated with emotion observation. A decrease in finger SkBF could indicate a sympathetic response (Kistler et al., 1998), showing that participants in our study were not in a high arousal state in response to observing others’ faces, including angry expressions with blushing. Therefore, based on previous study findings regarding finger SkBF, the significant decrease of facial SkBF in response to angry expressions with blushing was not explained by sympathetic nervous system predominance (such as fear of emotional faces). Facial SkBF is a robust or sensitive index related to anger, compared with finger SkBF and RR interval.

Concerning the ANS dynamics, the RR interval changes in all types of faces reflect parasympathetic nervous system (PNS) dominance (Cribbet et al., 2020). Based on the abovementioned mechanism, the regulation of facial SkBF and the RR interval is different, especially related to the observation of angry expressions with blushing. The heart rate reflects PNS dominance and the sympathetic nervous system induces facial vasoconstriction, as reported in previous studies (Kosonogov et al., 2017; Matsukawa et al., 2018). However, there is little consensus about the innervation of facial vessels. This could be a direction for future research.

### 4.3 Limitations and recommendations

Our study revealed that facial SkBF generally decreased in response to observing others’ emotional faces and significantly decreased in response to angry expressions with blushing. However, further studies are required, especially regarding the following four points:

First, a previous study reported that blood flow dynamics vary depending on the location of the face (Ioannou et al., 2014a). Previous studies measured facial SkBF in people experiencing emotions such as anger, embarrassment, or joy (Drummond, 1994, 1999; Drummond and Quah, 2001; Brummelman et al., 2018) and showed that facial SkBF could be an indicator of emotions. However, our study showed a decrease in facial SkBF in response to observing others’ emotions, indicating that facial SkBF is an emotional index even when observing others’ emotions. Based on reports that facial blood dynamics differ depending on the location of the face, further studies measuring facial SkBF in areas other than the cheek, such as the forehead, ears, or nose, are required to confirm the importance of facial SkBF in the observation of emotions.

Second, in this experiment, participants were unaware of blushing in facial stimuli. Facial SkBF significantly decreased in response to observing a combination of explicitly perceived angry expressions and implicitly perceived facial blushing. However, there are several possibilities regarding the dynamics of facial SkBF associated with observing others’ emotions. For instance, observing angry faces with blushing may induce a more significant decrease in facial SkBF when the observers are explicitly aware of the other’s blushing than when they are unaware. Moreover, the stronger the level of facial blushing in others, the more significant the decrease in the observers’ facial SkBF might be.

Third, our results showed a decrease in facial SkBF, which may induce new interactions. A decrease in facial SkBF can induce perceptible pallor, which can influence the one displaying intense emotions or a new third party. Therefore, it is necessary to examine whether the decrease in facial SkBF associated with observing angry facial expressions with blushing is reflected in the facial colour of the observer and whether facial pallor causes new interactions.

Finally, the facial SkBF change associated with observing others’ expressions, as shown by our study, reflects the blood flow dynamics of healthy participants and thus may apply to diseases or developmental disorders related to facial SkBF, such as erythrophobia. Comparing facial SkBF associated with observing others’ emotions between healthy people and people with the abovementioned disorder might lead to the elucidation of their mental problems from the perspective of facial SkBF.

There are several limitations to this study. We asked participants to refrain from ingesting vasoactive substances 3 h before the experiment (Ioannou et al., 2017). However, the effects of vasoactive substances may persist for more than 3 h. Another limitation would be that, in our experiment, participants observed only angry and embarrassed expressions, which are negative emotions. We chose these two because previous studies have shown that anger and embarrassment greatly increase facial SkBF (Shearn et al., 1990; Drummond, 1999), but we need to experiment with positive facial expressions in the future.

## 5 Conclusion

The dynamics of facial SkBF in the ANS depend largely on psychological factors, but its underlying mechanisms and relationships with other autonomic indices remain ambiguous. Our results show the sensitivity and reactivity of facial SkBF in reaction to the perception of emotional faces, especially angry faces with blushing, compared with finger SkBF and cardiac indices. We also provide a perspective on the emotional observers’ facial SkBF in addition to that of the people experiencing emotions.

Our study expands our understanding of the mechanisms and functions of facial SkBF in emotional interactions and clinical applications to diseases related to facial temperature and colour in terms of typical facial SkBF dynamics or function.

## Data availability statement

Data supporting the findings of this study are available on request.

## Ethics statement

The studies involving humans were approved by the Research Ethics Committee of the Faculty of Letters, the Graduate School of Faculty, and the Graduate School of Social Science of Keio University. The studies were conducted in accordance with the local legislation and institutional requirements. The participants provided their written informed consent to participate in this study. Written informed consent was obtained from the individual(s) for the publication of any potentially identifiable images or data included in this article.

## Author contributions

NI: Conceptualization, Data curation, Formal analysis, Investigation, Software, Visualization, Writing – original draft. MA: Methodology, Validation, Writing – review & editing, Formal analysis. SU: Funding acquisition, Supervision, Writing – review & editing, Formal analysis, Investigation, Project administration.
